# Dosimetric Impact of Voluntary Deep Inspiration Breath Hold (DIBH) in Mediastinal Hodgkin Lymphomas: A Comparative Evaluation of Three Different Intensity Modulated Radiation Therapy (IMRT) Delivery Methods Using Voluntary DIBH and Free Breathing Techniques

**DOI:** 10.3390/cancers16040690

**Published:** 2024-02-06

**Authors:** Samarpita Mohanty, Divya Patil, Kishore Joshi, Poonam Gamre, Ajay Mishra, Sunil Khairnar, Sangeeta Kakoti, Lingaraj Nayak, Sachin Punatar, Jeevanshu Jain, Reena Phurailatpam, Jayant S. Goda

**Affiliations:** 1 Department of Radiation Oncology, Tata Memorial Centre, Homi Bhabha National Institute, Mumbai 410210, India; samarpita.mohanty@gmail.com (S.M.); patildivya51@gmail.com (D.P.); kj61288@gmail.com (K.J.); poonamgamre@gmail.com (P.G.); ajay291176@rediffmail.com (A.M.); sunil1308@rediffmail.com (S.K.); drsangeeta84@gmail.com (S.K.); jeevs.jn@gmail.com (J.J.); reena.ph@gmail.com (R.P.); 2 Department of Hemato Oncology, Tata Memorial Centre, Homi Bhabha National Institute, Mumbai 410210, India; lingarajnayak86@gmail.com (L.N.); drsachin_punatar@yahoo.in (S.P.)

**Keywords:** voluntary deep inspiration breath hold, mediastinal Hodgkin lymphoma, dosimetry, full volumetric arc therapy, butterfly VMAT, intensity modulated radiotherapy

## Abstract

**Simple Summary:**

Radiation is usually used as a consolidation in early-stage mediastinal Hodgkin lymphomas with excellent cure rates and long-term survival. Patients undergoing mediastinal radiotherapy for Hodgkin lymphoma have a risk of serious radiation-associated late toxicities such as cardiovascular disease and secondary cancers. Historically, mediastinal Hodgkin lymphomas used to be treated via simple conventional AP-PA or 3D-CRT in free breathing. The advent of IMRT and deep inspiration breath hold (DIBH) techniques have allowed more conformal treatment with reduced doses of the organs at risk (OARs). We explored the impact of these techniques in limiting the irradiation of the lungs, heart, and breast without compromising the dose to the target. Plans were calculated using three different IMRT modalities with and without DIBH. The adoption of DIBH resulted in a significantly reduced radiation dose to the OARs for all the IMRT delivery techniques compared with free breathing.

**Abstract:**

Hodgkin lymphomas are radiosensitive and curable tumors that often involve the mediastinum. However, the application of radiation therapy to the mediastinum is associated with late effects including cardiac and pulmonary toxicities and secondary cancers. The adoption of conformal IMRT and deep inspiration breath- hold (DIBH) can reduce the dose to healthy normal tissues (lungs, heart and breast). We compared the dosimetry of organs at risk (OARs) using different IMRT techniques for two breathing conditions, i.e., deep inspiration breath hold (DIBH) and free breathing. Twenty-three patients with early-stage mediastinal Hodgkin lymphomas were accrued in the prospective study. The patients were given treatment plans which utilized full arc volumetric modulated arc therapy (F-VMAT), Butterfly VMAT (B-VMAT), and fixed field IMRT (FF-IMRT) techniques for both DIBH and free breathing methods, respectively. All the plans were optimized to deliver 95% of the prescription dose which was 25.2 Gy to 95% of the PTV volume. The mean dose and standard error of the mean for each OAR, conformity index (CI), and homogeneity index (HI) for the target using the three planning techniques were calculated and compared using Student’s *t*-test for parametric data and Wilcoxon signed-rank test for non-parametric data. The HI and CI of the target was not compromised using the DIBH technique for mediastinal lymphomas. The mean values of CI and HI for both DIBH and FB were comparable. The mean heart doses were reduced by 2.1 Gy, 2.54 Gy, and 2.38 Gy in DIBH compared to FB for the F-VMAT, B-VMAT, and IMRT techniques, respectively. There was a significant reduction in V5Gy, V10Gy, and V15Gy to the heart (*p* < 0.005) with DIBH. DIBH reduced the mean dose to the total lung by 1.19 Gy, 1.47 Gy, and 1.3 Gy, respectively. Among the 14 female patients, there was a reduction in the mean right breast dose with DIBH compared to FB (4.47 Gy vs. 3.63 Gy, *p* = 0.004). DIBH results in lower heart, lung, and breast doses than free breathing in mediastinal Hodgkin Lymphoma. Among the different IMRT techniques, FF-IMRT, B-VMAT, and F-VMAT showed similar PTV coverage, with similar conformity and homogeneity indices. However, the time taken for FF-IMRT was much longer than for the F-VMAT and B-VMAT techniques for both breathing methods. B-VMAT and F-VMAT emerged as the optimal planning techniques able to achieve the best target coverage and lower doses to the OARs, with less time required to deliver the prescribed dose.

## 1. Introduction

Radiotherapy plays a crucial role as a part of multimodality treatment for mediastinal Hodgkin lymphoma [1]. Hodgkin lymphoma is a highly radiosensitive neoplasm that often involves the mediastinum. The application of radiotherapy to the mediastinum poses challenges due to the complex target volume, the proximity of the organs at risk (OARs) like the lungs and the heart, and respiratory motion. These factors can result in increased doses to the heart and lungs leading to late toxicities particularly cardiovascular diseases and second malignancies significantly affecting the patient’s quality of life [2,3,4].

To reduce the toxicities associated with treatment, radiotherapy for Hodgkin lymphoma has evolved in terms of the treatment volume, dose, and technique. The radiotherapy volume has shifted from extended field irradiation (such as mantle field irradiation) and involved field radiation therapy (IFRT) to involved site radiation therapy (ISRT) and involved node radiation therapy (INRT) with the evolution in sophisticated radiation delivery technologies [5,6]. Additionally, with the advent of highly active chemotherapeutic agents, the standard of care for treating early-stage Hodgkin lymphoma has undergone a paradigm shift in the form of shortened chemotherapy and a reduction in volumes with lower radiotherapy doses [5,7]. Traditionally, two anterior–posterior and posterior–anterior radiation beams were used to treat mediastinal lymphoma to reduce lung doses. Over the years, radiotherapy techniques have evolved from conventional two-dimensional (2D) radiation to conformal techniques such as intensity-modulated radiation therapy (IMRT) and volumetric modulated arc therapy (VMAT) [8,9]. However, IMRT results in larger volumes being exposed to lower doses, especially lungs and breasts. Dedicated techniques such as “butterfly” volumetric modulated arc therapy (B-VMAT) have been implemented using a unique beam configuration to lower the exposure to organs such as the breast and lungs [10]. Various other authors have also proved the efficacy of various IMRT or VMAT techniques for mediastinal lymphomas [11,12].

These advanced methods have the advantage of increasing the likelihood of delivering a conformal dose to the target volume while reducing the dose to OARs, but the effects of intrafraction tumor mobility, mainly due to respiratory motion, pose a real challenge as they result in a complex dose distribution with hot and cold spots and pose the risk of missing the target due to the increasing practice of keeping conservative margins for the fear of dose spillage to the surrounding OARs. Secondly, the interplay effect caused by the combination of the beam motion (as it is shaped by the dynamic multi-leaf collimators) and the changing amplitude and frequency of respiratory motion results in the complex dose distribution having hot and cold spots within the target [13,14]. Therefore, adopting the breath hold method with IMRT delivery techniques in lymphomas in the mediastinum may offset the uncertainties caused by the respiratory motion, thereby improving target coverage and simultaneously reducing the doses to normal tissues.

Deep inspiration breath hold (DIBH) is a unique method that has emerged as a motion management method that minimizes motion-induced dose variations by having patients hold their breath during treatment delivery. Additionally, it allows for a reduction in doses to the lungs and heart by increasing the lung volume and displacing the heart inferiorly, respectively, and has provided benefits in treating left-sided breast cancer [15,16]. In mediastinal lymphomas, a prospective phase II study has shown a lower mean lung and heart dose with DIBH compared to free breathing (FB) [17,18].

Studies using the DIBH breathing method in mediastinal lymphomas have compared IMRT (VMAT) with 3D CRT or B-VMAT (the butterfly technique) with full VMAT. However, studies comparing various radiotherapy delivery techniques (i.e., full arc VMAT, butterfly VMAT, and fixed field IMRT) for both breathing methods are limited [12]. In this study, we compared the effectiveness of two breathing methods, DIBH and FB, in treating mediastinal lymphoma using fixed field IMRT (FF-IMRT), full arc VMAT (F-VMAT), and butterfly VMAT (B-VMAT) to optimize the best delivery technique while using these two methods for the ease of future implementation in clinical practice.

## 2. Materials and Methods

**Figure 1:**
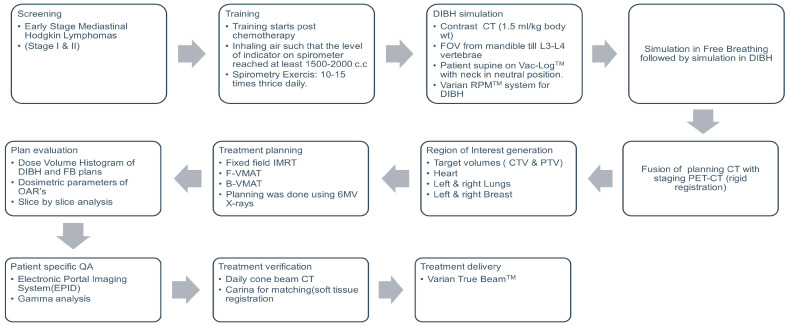
The workflow for patient treatment for mediastinal Hodgkin Lymphoma in a radiotherapy department. DIBH: Deep Inspirational Breath Hold; RPM: Real-time position management; IMRT: Intensity Modulated Radiotherapy; OAR: Organs at risk; F-VMAT: Full arc Volumetric Modulated Arc therapy; B-VMAT: Butterfly Volumetric Modulated Arc Therapy; CTV: Clinical Target Volume; PTV: Planning Target Volume; ROI: Region of Interest; CT: Computed Tomography; PET: Positron Emission Tomography; FOV: Field of View.

The study was a prospective observational study approved by the Institutional Ethics Committee (IEC-1979). Patients with a diagnosis of early-stage mediastinal Hodgkin lymphoma (stage I and II) were included in the study. Patients with a mediastinal disease with extension to the supraclavicular neck nodes were included in the study. Patients with involvement of neck nodes above the supraclavicular nodal level (upper neck involvement) were excluded from the study. Figure 1 shows the workflow for patient treatment for mediastinal lymphoma using the DIBH method.

### 2.1. Patient Selection

Patients were screened at least 6 weeks before the start of radiotherapy. To be included in the study, the patient should have been able to hold the breath comfortably for at least 15–20 s in deep inspiration. If the principal investigator was satisfied with the patient’s breath hold duration, he or she was given instructions for proper training.

### 2.2. DIBH Training and Simulation

For the training in breath holding, the patient was given a spirometer. The training consisted of inhaling air so that the level of the indicator reached at least 2000 c.c. The patient was instructed to continue this exercise 10–15 times thrice daily. While training, the patient was asked to maintain a chart that was scrutinized by the principal investigator. If the indicator level on the spirometer did not reach the requisite level, corrective measures were taken by the investigator which included training the patients in deep breathing exercises. The deep breathing exercises eventually helped the patient to improve the depth of inspiration and also increased the breath hold time without any discomfort. None of the patients had to be rescanned during therapy as they were able to hold their breath comfortably.

On the day of the simulation, a radiation therapist (RTT) gave the patients instructions to hold their breath for 15–20 s before the simulation to prepare for a DIBH scan. A patient’s ability to hold their breath in the treatment position was assessed over two to three practice sessions. The free breathing scan was obtained followed by a DIBH scan with intravenous contrast (1.5 mL/kg) conducted on the same day. The patient was immobilized in a supine position with the neck in a neutral position on a Vac-Loc™ with the arms above the head. A trace of the respiratory pattern for the patient was obtained using Varian Real-time Position Management (RPM V.1.8). A CT scan was acquired from the mandible to the bottom of the L2 vertebrae.

### 2.3. Contouring

Target and OAR delineations were performed using the Varian Eclipse (Varian Medical Systems V.16.1) contouring software. The pre- and post-chemotherapy PET scans, which were conducted under FB conditions, were fused with the simulation CT scan. Pre-chemo and post-chemo clinical target volumes (CTVs) and OARs in proximity were generated as per the International Lymphoma Radiation Oncology Group (ILROG) guidelines on both DIBH and FB scans [5,19].

### 2.4. Treatment Planning

For each simulation technique, DIBH and FB, three plans of fixed-field IMRT(FF-IMRT), full arc VMAT (F-VMAT), and butterfly VMAT (B-VMAT) were created using the Varian Eclipse™ Treatment Planning System (V.16.1). The Photon Optimizer (V.16.1) and the Acuros XB algorithm (V.16.1) were used to optimize and compute the plans. The FF-IMRT plans were planned with 5–7 static fields according to the target volume, shape, and proximity of OARs with parallel opposing techniques. The F-VMAT plans were generated using two full arcs (360°) with 6MV energy. Two coplanar arcs with 50–60° avoidance sectors were used to plan B-VMAT plans to spare the lungs. All the plans were optimized and normalized so that 95% of the PTV volume received 95% of the prescription dose. The conformity index and homogeneity index were calculated and compared between the DIBH and the FB methods [20,21]. The treatment plan deliverability, the total monitor unit, the complexity index, and the gamma passing rate were also evaluated. To estimate the complexity index of a given plan (i.e., the rate at which the fluence varied across each field), the Eclipse Scripting Application Programming Interface (ESAPI) script in the Eclipse™ treatment planning system version 16.1 was used [22]. The plans were evaluated and approved by a senior radiation oncologist (with experience of more than 15 years). To check the deliverability, patient-specific quality assurance was carried out using an electronic portal imaging device (EPID).

### 2.5. Treatment and Verification

A Varian true beam linear accelerator (V.2.7) was used to treat the patients with Varian RPM for motion management. The upper and lower thresholds for DIBH were set according to individual patient breathing waveforms with a 0.5 mm window and 2 s delay in the breathing pattern for turning the beam on. Daily cone beam CT (CBCT) was used for treatment verification. The CBCT was matched using the carina and vertebrae as landmarks to determine whether the patient was positioned properly. While verifying the registration, we ensured that the PTV was also covered adequately.

### 2.6. Data Collection and Analysis

PTV coverage and dosages to lungs (left, right, and total), breast (left and right), heart, and integral dose to the non-target body were recorded, and IBM SPSS (V. 26) software was employed for statistical analysis. The integral dose to the non-target body was calculated as ID [Gy⋅cc] = D [Gy]⋅V [cc], where D [Gy] is the mean dose delivered to volume V [cc] (where cc is cm^3^) [23]. The Shapiro–Wilk’s test was used to check the normality of the dataset. If the Shapiro–Wilk’s test was non-significant (*p* > 0.05), the data were considered to be normally distributed, and a parametric test, i.e., a one-way ANOVA test, was used to compare all the three different treatment groups (B-VMAT, F-VMAT and FF-IMRT) techniques. If the Shapiro–Wilk’s test was significant (*p* < 0.05), the distribution of the data was not considered to be normal, and then a non-parametric Kruskal–Wallis test was used (Supplementary Tables S2A and S2B). The dosimetric indices of the target volumes (PTVs) and the doses to the organ at risk (OARs) for the two breathing conditions (DIBH and FB) were compared using the Wilcoxon signed-rank test for non-parametric data and Student’s *t*-test for parametric data.

## 3. Results

### 3.1. Demographic and Treatment Profile of Patients

This prospective study included twenty-three patients affected by early-stage lymphoma treated at our center from 2018 to 2021 with radiotherapy at the mediastinal site using the DIBH-IMRT technique. The median age of the patients was twenty-seven years (Interquartile range, 21–31.5), and fourteen among them were females. The prescribed dose of radiotherapy to the mediastinum in all the plans was 25.2 Gy in 14 fractions based on the institutional policy (patients who had complete metabolic response with Deauville scores of 1, 2, and 3) on interim PET were consolidated with a radiation dose of 25.2 Gy in 14 fractions, while patients who had a partial response (a Deauville score of4) were consolidated with a radiation dose of 34.2 Gy in 19 fractions. While the majority of the patients (*n* = 21) were treated with the B-VMAT technique, two patients were treated with F-VMAT and FF-IMRT delivery techniques, respectively. The patient characteristics and radiotherapy details are listed in Table 1.

### 3.2. Volumes of PTV and OARs

The effect of DIBH on the PTV and OAR volumes is depicted in Table 2. The PTV was slightly smaller with DIBH (mean: 537.73 c.c.) vis-à-vis the PTV in FB (mean: 556.97 c.c.). The mean difference in the PTV volumes in DIBH compared to FB was not statistically significant (*p* = 0.059). However, the DIBH technique resulted in significant differences in the volume of OARs, especially in the lungs and the heart. While the mean lung volume in the DIBH scan was 3598.62 c.c., the mean lung volume was 2131.1 c.c. in the FB scan, resulting in a 68.9% increase in its volume with DIBH (*p* < 0.001). Compared to FB, the DIBH technique resulted in a statistically significant smaller mean heart volume (504.4 c.c. vs. 444 c.c., *p* < 0.001).

### 3.3. Dosimetric Analysis

**Figure 2:**
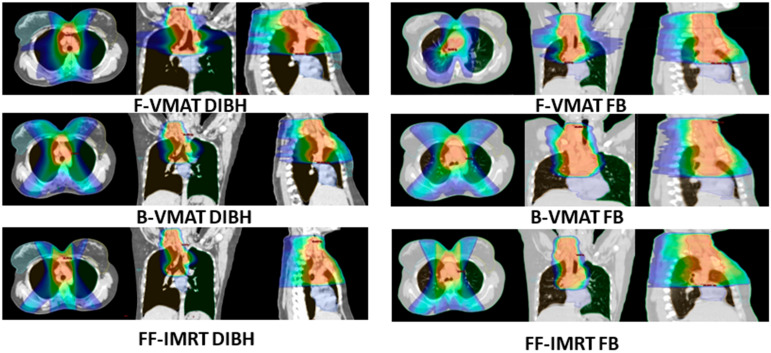
Dose color wash of the representative patient in axial, coronal sagittal CT images regarding three planning techniques used alongside DIBH and FB breathing methods. The volume receiving 5 Gy is indicated by blue color The green color wash represents the volume receiving 17.5 Gy (70%), while the orange color dose wash represents the volume receiving 23.9 Gy (95%). F-VMAT: full arc-volumetric modulated arc therapy; B-VMAT: butterfly volumetric modulated arc therapy: FF-IMRT: fixed field intensity-modulated radiation therapy; DIBH: deep inspiration breath hold; FB: free breathing.

Figure 2 depicts the representative dose color wash achieved for a patient with mediastinal Hodgkin lymphoma using the three planning techniques (F-VMAT, B-VMAT and FF-IMRT) and the two breathing conditions (DIBH and FB) with a prescribed dose of 25.2 Gy. The blue color wash represents the volume receiving 5 Gy (20%), the green color wash represents the volume receiving 17.5 Gy (70%), while the orange color dose wash represents the volume receiving 23.9 Gy (95%).

#### 3.3.1. Plan Quality and Deliverability

Table 3 shows comparisons of the plan quality in terms of the target volume coverage with both DIBH and FB among the three techniques. In all the plans, 95% of the PTV received at least 95% of the prescribed doses. There were no statistically significant differences in the PTV coverage between the plans. The homogeneity and conformity indices were not significantly different between the two plans.

As indicated in Table 3, while there were no significant differences in the monitor units between the planning techniques utilizing DIBH and FB, FF-IMRT resulted in higher monitor units than F-VMAT and B-VMAT. Gamma analysis with EPID was used to evaluate the deliverability of all plans. With a 3%/3 mm criterion and 10% threshold, all of the plans displayed gamma passing rates higher than 95%. To rule out the over modulation of plans, the complexity was compared. The complexity was in the range of 0.1410 and 0.1625, and no significant difference was observed between DIBH and FB.

#### 3.3.2. Doses to OARs

**Figure 3:**
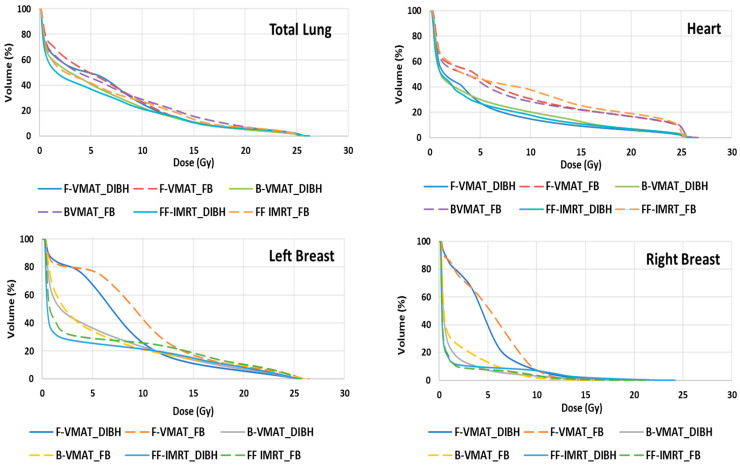
Comparison of dose–volume histograms for DIBH and FB simulation techniques for FVMAT, B-VMAT and FF IMRT plans. F-VMAT: full arc volumetric modulated arc therapy; B-VMAT: butterfly volumetric modulated arc therapy; FF-IMRT: fixed-field intensity-modulated radiation therapy; DIBH: deep inspiration breath hold; FB: free breathing. The dotted line represents the FB condition, and the solid line represents the DIBH breathing condition.

Table 4 shows the achieved dose parameters and the absolute and relative differences in the doses for the OARs for each of the six plans. A dose–volume histogram (DVH) for DIBH and FB breathing conditions for the three radiotherapy delivery techniques, F-VMAT, B-VMAT, and FF-IMRT, were compared and are shown in Figure 3. The DVH showed favorable dosimetry for all the OARs (lung, breast, and heart) when planned with the DIBH method irrespective of the radiotherapy delivery technique used. As observed from the DVH curves in Figure 3, the reduction in radiation doses to the heart with DIBH was quite conspicuous irrespective of the radiation delivery technique used.

#### 3.3.3. Heart

The mean cardiac doses were significantly lower with DIBH compared to FB. The radiotherapy dose was reduced by 2.1 Gy (29.7%), 2.5 Gy (33.4%), and 2.38 Gy (29.3%) in the F-VMAT, B-VMAT, and FF-IMRT techniques, respectively. With DIBH, there was a significant decline in the cardiac volume, receiving 5 Gy, 10 Gy, and 15 Gy (*p* < 0.05). The reduction in the mean cardiac dose varied significantly among individual patients. The individual variation in the mean heart dose was due to the variation in the proximity of the target volume to the heart, with target volumes confined to the superior mediastinum (away from the heart), showing a greater reduction in heart doses.

#### 3.3.4. Lungs

The reduction in the mean dose to the lungs using the DIBH method is dependent on the overlap between the PTV and the lung along with the degree of the expansion of the lungs. The total lung volume in the DIBH method increased by approximately 1500 c.c. when compared to FB (*p* < 0.05). There was significant variability in percentage differences in the mean lung dose (Gy), as represented in Table 4. The three radiotherapy delivery techniques (F-VMAT, B-VMAT and FF-IMRT) using DIBH methods reduced the mean lung dose by 1.19 Gy (17.2%), 1.47 Gy (20.9%), and 1.3 Gy (19.5%), respectively, when compared to FB. Additionally, the DIBH method resulted in lower V5Gy, V10Gy, and V25Gy lung doses. The B-VMAT and FF-IMRT technique when combined with DIBH was able to achieve the lowest V5Gy to the lungs.

#### 3.3.5. Breast

Among the fourteen female patients, the reduction in the mean dose to the right breast was significant with DIBH compared to FB for BVMAT (4.47 Gy vs. 3.63 Gy, *p* = 0.004) but non-significant for VMAT and IMRT techniques. Additionally, the DIBH method resulted in reduced V10Gy to both breasts using FVMAT and BVMAT techniques.

#### 3.3.6. Comparison of Three Different Radiotherapy Delivery Techniques

F-VMAT, B-VMAT, and FF-IMRT plans were calculated for all 23 consecutive patients with mediastinal Hodgkin lymphomas in both FB and DIBH scans. The planning target volume V 95% was kept comparable between all plans while reducing organ doses as much as possible.

The FB F-VMAT plans showed the best homogeneity index (HI = 0.086), followed by DIBH F-VMAT (HI: 0.089), while others (B-VMAT and FF-IMRT for both breathing methods) showed a HI index in the range of 0.090–0.097. Upon applying the statistical test, no statistical significance was observed, as the magnitude of the differences was small between all the techniques. The FB F-VMAT plan showed the highest conformity (CI = 1.014), followed by the DIBH F-VMAT plan (1.017), the DIBH B-VMAT plan (CI: 1.034), and the FB B-VMAT (CI: 1.048). The FB FF-IMRT plan had a CI of 1.087, and finally, the DIBH FF-IMRT plan had a CI of 1.093. All differences were statistically insignificant (Table 3).

Upon comparing the plans achieved for reducing the doses to OARs via the three radiotherapy delivery techniques using DIBH, it was observed that V5Gy (%) of the left lung was reduced significantly with DIBH FF-IMRT (*p* = 0.012) compared to DIBH F-VMAT or DIBH B-VMAT. Similarly, V5Gy (%) of the right lung and V5Gy (%) of the total lung were reduced significantly with DIBH FF-IMRT (*p* = 0.032) and (*p* = 0.025), respectively. For the right breast, V5Gy (%) and V10Gy (%) were significantly reduced with DIBH F-VMAT compared to DIBH B-VMAT and DIBH FF-IMRT (*p* = 0.035 and *p* = 0.032), respectively. There was no difference in the dosimetric indices of other OARs (heart) when comparing F-VMAT, B-VMAT and FF-IMRT for the DIBH breathing condition (Supplementary Table S2A).

Upon comparing the plans achieved for reducing the doses to OARs via all three radiotherapy delivery techniques using the free breathing method, the FB FF-IMRT technique reduced the mean lung dose significantly compared to FB B-VMAT and FB F-VMAT (*p* = 0.017). On the contrary, FB FF-IMRT was able to significantly reduce the volume of the left breast receiving 4 Gy [V4Gy (%)] and 10 Gy [V10Gy (%)] compared to FB B-VMAT and FB F-VMAT (*p* = 0.015 and *p* = 0.003), respectively. There was no difference in the dosimetric indices of other OARs (heart) upon comparing F-VMAT, B-VMAT and FF-IMRT for the free breathing condition (Supplementary Table S2B).

## 4. Discussion

Modern radiotherapy techniques such as IMRT and VMAT along with motion management methods such as deep inspiration breath hold (DIBH) have revolutionized the treatment of mediastinal lymphoma, offering improved dose conformity to the target volume while reducing the doses to adjacent organs at risk (OARs) and potentially minimizing toxicities [10,11,17,18,19,24,25]. Our study supports the findings of previous studies that DIBH helps in reducing the doses of the OARs including the lungs and heart. Additionally, among the different radiotherapy techniques, full arc VMAT exhibited a statistically significant increased low-dose exposure to the lungs (V5Gy) and to the breasts (V4Gy).

Radiotherapy for mediastinal lymphoma remains challenging owing to the complex shape of the target volume close to the critical structures. Additionally, mediastinal lymphomas often occur in younger populations with potential long-term survivorship [2]. Hence, minimizing the late toxicities, especially in the younger population, remains crucial [4]. The volumes of radiotherapy in lymphomas have evolved from large field radiation like extended field RT (EFRT) to reduced field RT including involved field radiotherapy (IFRT), involved site radiotherapy (ISRT), and involved node radiotherapy (INRT) [1]. With the advancement in radiotherapy techniques, more conformal radiotherapy techniques including IMRT and VMAT have been adopted to improve the dose conformity to the target volume and decrease the doses to OARs.

While IMRT has improved target coverage and reduced heart doses, it has increased the volume of normal tissues exposed to radiation. To mitigate this issue, butterfly IMRT was developed by Fiandra et al. with limited beam angles to improve conformity to the target volume while reducing the low-dose bath to the lungs and breasts [12]. The dose distribution resembled a butterfly, hence the name. Later, the butterfly VMAT technique was developed using a unique beam configuration with two coplanar and one non-coplanar arc to achieve a similar dose distribution [10]. In our study, we used two coplanar arcs for ease of set-up and treatment delivery. FVMAT resulted in increased low-dose exposure to the lungs and to the breasts. This is consistent with findings from previous studies which have shown increased V5Gy to the lungs and breasts with F-VMAT [25].

Our analysis showed that DIBH resulted in a significant reduction in heart doses. There was a reduction in the mean heart dose ranging from 2.1 Gy to 2.5 Gy with the incorporation of DIBH. This decrease is a result of displacing the heart inferiorly away from the target volume with deep breathing. There was a significant reduction in V5Gy, V10Gy, and V15Gy to a range of 24–40% with the DIBH technique. Similar findings have been consistently reported in previous studies [17,25]. There was marked inter-patient variability in the difference in the mean heart dose achieved with various plans. While one patient experienced a substantial decrease of 8.59 Gy with DIBH, three other patients exhibited an average reduction in heart dose of around 0.5 Gy. This may likely be the result of differences in the proximity of the target volume to the heart.

Similarly, there have been reports of reduced lung doses with the incorporation of DIBH [17,18,25]. In our study, DIBH resulted in a decrease in the mean lung doses by 1–1.5 Gy. There was a 10% difference in V5Gy in both lungs with the BVMAT technique with the use of DIBH. There have been conflicting reports on the effect of DIBH on doses to breasts. Our analysis showed that DIBH resulted in a reduction in the mean right breast dose of0.84Gy (*p* = 0.004) with the B-VMAT technique, while V10Gy was reduced with both F-VMAT and BVMAT. This is similar to the findings reported by Starke et al. [25]. This may have resulted from the breasts moving away from the target volume laterally during deep inspiration. However, Houlihan et al. reported an increase in the mean breast dose of 0.6 Gy with DIBH [17]. The discordant results are probably due to interpatient variability in the anatomy of breasts. In our study, F-VMAT resulted in increased low-dose exposure (V4Gy) to both the breasts and lungs (V5Gy) compared to FF-IMRT and B-VMAT techniques (Supplementary Table S1). Similar findings have been reported in other studies as well [25].

The volume of normal tissue exposed to a low radiation dose has an impact on late complications like second malignancies, while intermediate and high-dose exposure has an impact on early complications like radiation pneumonitis. Epidemiological studies from large cohorts of HL patients showed that traditional RT volumes and radiation doses were associated with long-term toxicities like second malignancies and cardiac morbidity [3,26,27,28]. Traditional mathematical models (Lymen–Burman–Kutsher and logistic regression models) are being used by clinicians to estimate the risk of long-term morbidity from the data obtained from patients treated with conventional RT techniques from small prospective and retrospective clinical studies [29,30,31]. However, these techniques are no longer being used in the contemporary management of mediastinal Hodgkin lymphoma. Therefore, the risk estimates calculated from these models may not have relevance in the contemporary era where highly conformal radiation delivery is practiced. Moreover, these models suffer from the limitations of using binary outcomes and DVH parameters [30,31], making them over simplistic as they do not take into consideration the actual heterogeneity of the dose distribution and the anatomical complexity of the tissues and organs. These models are developed on different mathematical and biological assumptions, and the robustness of one model over the other is still debatable. Till the time clinically relevant models are developed that can predict the long-term morbidities of these techniques with some amount of accuracy, more mature clinical data have to be generated to understand the actual impact of these highly conformal techniques on long-term complications of RT given through these techniques. Due to the lack of data on long-term toxicities with newer techniques, only dosimetric metrics are being used by clinicians to investigate the impact of conformal and IMRT techniques with DIBH [9,10,12,17]. Since the radiation doses received by a certain volume of normal tissue can be a surrogate for either early or late toxicity, our study investigated the dosimetry of all three conformal radiation delivery techniques with the DIBH method in terms of normal tissue volumes receiving low and intermediate doses. We observed that F-VMAT and B-VMAT reduced the intermediate dose exposure to both breasts compared to FF-IMRT (Right breast V10 Gy (%) was 8.19% with F-VMAT, 12.13% with B-VMAT and 15.78% with FF-IMRT). For the left breast, V10Gy (%) was 13.16% with F-VMAT,14.97% with B-VMAT, and 20.42% with FF-IMRT (Supplementary Table S1). However, there was not much difference in intermediate dose exposure to the lungs and heart with any of the three techniques using the deep inspiration breath hold method (Supplementary Table S1). When the three radiotherapy techniques were evaluated for FB, the % volume of both the right and left breast receiving 10 Gy (V10Gy) was similar to F-VMAT, B-VMAT and FF-IMRT (Supplementary Table S1).

DIBH resulted in a significant increase (69%) in the volume of the lungs. At the same time, there was a significant reduction in the mean heart volume which may have been the result of a compression of the heart due to deep breathing. This finding has been consistent across various studies corroborating our observations [17,25]. The PTV volumes have been reported to be smaller with DIBH in the study by Starke et al. [25]. This is likely due to the differential margins used in the different breathing methods. We report no difference in the PTV volumes, as the CTV to PTV margins were the same with both breathing conditions (DIBH vs. FB). There were no statistically significant differences in the homogeneity and conformity indices between the different plans. These findings differ from the results reported by Starke et al. where B-VMAT plans yielded less plan homogeneity due to hotspots distributed mostly around the sternum [25]. In our study, FF-IMRT plans resulted in the delivery of higher MUs and thus a higher treatment time than VMAT plans, as expected. Hence, from a practical perspective, one of the merits of VMAT lies in its ability to efficiently administer complex IMRT treatment with swift delivery. In our study, while there was a statistically significant increase in monitor units (MUs) when employing DIBH compared to FB in the B-VMAT technique, this difference lacked clinical significance.

We compared the plans achieved with all three IMRT techniques for both breathing methods studied. The target coverage was similar with all three techniques, and so were the HI and CI with insignificant differences between the techniques (Table 3). With regards to the dosimetry of the OARs, low-dose spillage in the lungs and breasts was significantly higher with F-VMAT, followed by B-VMAT, and least with FF-IMRT (Supplementary Tables S2A and S2B). However, the volumes of lung tissue and breast tissue receiving intermediate and high doses were nearly similar with all three techniques. For the heart, we did not observe any significant difference in the volume of the heart receiving a low dose or high dose (Supplementary Tables S2A and S2B) with the three techniques. The fact that no statistically significant results were obtained is at least partially likely due to the anatomic variability of the disease and patients.

Based on our observations, all the different IMRT techniques (FF-IMRT, B-VMAT, and F-VMAT) showed similar PTV coverage, conformity, and homogeneity indices for both breathing methods (DIBH or FB). However, the time taken by the FF-IMRT technique was much longer than the F-VMAT and B-VMAT techniques for both breathing methods. This may have implications on treatment compliance as the patient has to remain immobile for a longer duration, compromising the patient’s comfort.

B-VMAT and FVMAT emerged as the optimal planning techniques, able to achieve the best target coverage and lower doses to the OARs with less time required to deliver the prescribed dose.

Our study has a few limitations. Though it demonstrates the dosimetric parameters with different radiotherapy planning techniques and breathing methods, their impact on the outcomes and toxicities is not reported. Additionally, establishing a direct correlation between dosimetry and clinical outcomes, particularly in the context of long-term and uncommon toxicities, poses a challenge. Although multiple dosimetric studies and mathematical models have been developed to predict long-term complications like second malignancies, none of them have been validated clinically to date [9,17,18,25,32]. Secondly, the differences in the impact of the location of the target volume (upper vs. lower mediastinum) on the radiotherapy techniques have not been analyzed systematically. However, this study remains one of the few studies reported in the literature comparing the dosimetric parameters of different IMRT techniques with two different breathing conditions (DIBH and FB) in mediastinal Hodgkin lymphoma.

## 5. Conclusions

Deep inspiration breath hold offers significant advantages in terms of better sparing of the lungs and the heart in mediastinal Hodgkin lymphoma. Treatment planning using full arc VMAT results in a higher low-dose spillage to the lungs and the breasts as opposed to B-VMAT. Therefore, the choice of radiotherapy delivery technique should be carefully chosen based on patient characteristics and anatomical variation. Additional research is required to assess if proton beam therapy has further benefits in reducing the doses to the critical organs, especially in patients with lower mediastinal disease. Prospective studies should be planned to study the impact of advanced radiotherapy techniques on improving the toxicity profile of patients with mediastinal lymphoma.

## Figures and Tables

**Table 1:** Demographic and radiotherapy treatment details of mediastinal Hodgkin lymphomas.

Characteristics	Number of Patients (*n* = 23)
Median (range) age in years	27 (18–48)
Gender	
Males	9
Females	14
Planned dose	25.2 Gy
Planned no. of fractions	14
Planned dose per fraction	1.8 Gy
Technique used for treatment	
F-VMAT	1
B-VMAT	21
FF-IMRT	1

F-VMAT: full arc volumetric modulated arc therapy; B-VMAT: butterfly volumetric modulated arc therapy: FF-IMRT: fixed-field intensity-modulated radiation therapy; DIBH: deep inspiration breathhold; FB: free breathing.

**Table 2:** Comparison between planning target volume (PTV) and OAR volume between DIBH and free breathing modalities.

Target (PTV) and OAR Volumes	DIBH		FB		
	Mean (c.c.)	SEM	Mean (c.c.)	SEM	*p*-Value
PTV Volume	537.73	36.33	556.97	33.79	0.059
Left Lung Volume	1776.90	115.80	1047.78	73.85	0.000
Right Lung Volume	1993.07	129.96	1232.27	85.33	0.000
Total Lungs Volume	3771.21	244.63	2280.43	157.50	0.000
Heart Volume	444.03	28.58	504.42	32.51	0.000

DIBH; Deep inspiration breath hold; FB: free breathing.

**Table 3:** Dosimetry of target volume and deliverability of plans.

		F-VMAT	Absolute Difference (Gy)	Relative Difference (%)	*p*-Value	B-VMAT	Absolute Difference (Gy)	Relative Difference (%)	*p*-Value	FF-IMRT	Absolute Difference (Gy)	Relative Difference (%)	*p*-Value
PTV coverage	DIBH	95.62 ± 0.06	0.090	0.094	0.183	95.44 ± 0.06	0.15	0.16	0.117	95.67 ± 0.06	0.01	0.01	0.90
	FB	95.71 ± 0.07				95.59 ± 0.07				95.68 ± 0.06			
Homogeneity index (HI)	DIBH	0.089 ± 0.0017	0.007	0	0.324	0.097 ± 0.002	0.01	−4.74	0.143	0.090 ± 0.003	−0.0038	−4.42	0.20
	FB	0.0869 ± 0.0023				0.093 ± 0.002				0.09 ± 0.002			
Conformity index (CI)	DIBH	1.017 ± 0.004	0.01	0.99	0.505	1.034 ± 0.007	0.02	1.30	0.073	1.093 ± 0.007	−0.0066	−0.61	0.52
	FB	1.014 ± 0.004				1.048 ± 0.005				1.087 ± 0.010			
Monitoring units (MUs)	DIBH	635 ± 27	20	3.25	0.464	586 ± 19	61	−11.62	**0.013**	1109 ± 46	17	1.51	0.90
	FB	615 ± 16				525 ± 18				1126 ± 54			
Complexity	DIBH	0.1632 ± 0.0073	−0.0030	−2.33	0.640	0.1517 ± 0.0062	−0.0106	−7.59	0.171	0.1625 ± 0.0056	−0.0087	−5.64	0.17
	FB	0.1595 ± 0.0045				0.1410 ± 0.0044				0.1538 ± 0.0057			
Gamma passing rate (GPR)	DIBH	99.37 ± 0.12	0.15	−5.74	0.314	99.39 ± 0.14	0.48	−2.86	0.304	99.43 ± 0.14	−0.11	−4.68	0.352
	FB	99.52 ± 0.38				99.87 ± 0.44				99.32 ± 0.15			

The bold parameters indicate the data is statistically significant (*p*-value < 0.05).

**Table 4:** Dosimetry of organs at risk.

			F-VMAT				B-VMAT				FF-IMRT			
				Absolute Difference (Gy)	Relative Difference (%)	*p*-Value		Absolute Difference (Gy)	Relative Difference (%)	*p*-Value		Absolute Difference (Gy)	**Relative** **Difference (%)**	***p*** **-Value**
LUNG Left	DIBH	Mean (Gy)	5.64 ± 0.27	1.090	16.196	**0.000**	5.57 ± 0.27	1.44	20.53	**0.000**	5.33 ± 0.3	1.21	18.47	**0.000**
	FB		6.73 ± 0.32				7.01 ± 0.36				6.53 ± 0.42			
	DIBH	V5Gy [%]	41.17 ± 8.72	5.770	12.292	**0.017**	37.98 ± 8.33	8.98	19.13	**0.000**	33.09 ± 2.04	7.72	18.92	**0.000**
	FB		46.94 ± 8.12				46.97 ± 1.9				40.81 ± 2.57			
	DIBH	V10Gy [%]	22.16 ± 1.84	5.983	21.259	**0.000**	22.77 ± 1.58	7.87	25.69	**0.000**	22.43 ± 1.46	5.11	18.57	**0.001**
	FB		28.14 ± 2.17				30.64 ± 2.08				27.54 ± 2.28			
	DIBH	V20Gy [%]	5.42 ± 0.51	1.86	25.55	**0.002**	5.85 ± 0.58	2.28	28.04	**0.001**	6.38 ± 0.67	2.27	26.24	**0.003**
	FB		7.28 ± 0.87				8.13 ± 1.0				8.65 ± 1.06			
	DIBH	V25Gy [%]	1.53 ± 0.15	0.666	30.277	**0.005**	1.57 ± 0.16	0.79	33.55	**0.000**	1.49 ± 0.18	0.42	21.98	**0.045**
	FB		2.2 ± 0.28				2.36 ± 0.28				1.91 ± 0.27			
LUNG Right	DIBH	Mean (Gy)	5.73 ± 0.27	1.270	18.134	**0.001**	5.59 ± 0.29	1.52	21.38	**0.000**	5.40 ± 0.33	1.38	20.38	**0.000**
	FB		7.01 ± 0.41				7.11 ± 0.45				6.78 ± 0.51			
	DIBH	V5Gy [%]	42.76 ± 2.23	9.442	18.088	**0.003**	38.08 ± 1.97	10.59	21.75	**0.000**	34.23 ± 2.49	8.79	20.43	**0.000**
	FB		52.20 ± 3.19				48.67 ± 3.04				43.01 ± 3.37			
	DIBH	V10Gy [%]	21.71 ± 1.52	5.491	20.185	**0.003**	22.49 ± 1.56	7.69	25.48	**0.000**	22.86 ± 1.57	6.24	21.43	**0.001**
	FB		27.21 ± 2.20				30.19 ± 2.34				29.10 ± 2.54			
	DIBH	V20Gy [%]	5.22 ± 0.46	1.81	25.75	**0.005**	5.74 ± 0.60	2.08	26.60	**0.004**	6.51 ± 0.63	2.22	25.43	**0.010**
	FB		7.03 ± 0.80				7.82 ± 0.93				8.73 ± 1.08			
	DIBH	V25Gy [%]	1.36 ± 0.13	0.725	34.859	**0.003**	1.43 ± 0.16	0.68	32.29	**0.001**	1.20 ± 0.14	0.46	27.59	**0.000**
	FB		2.08 ± 0.28				2.11 ± 0.26				1.66 ± 0.27			
LUNGS	DIBH	Mean (Gy)	5.69 ± 0.24	1.185	17.242	**0.000**	5.58 ± 0.24	1.47	20.88	**0.000**	5.36 ± 0.26	1.30	19.50	**0.000**
	FB		6.87 ± 0.33				7.05 ± 0.35				6.66 ± 0.40			
	DIBH	V5Gy [%]	41.31 ± 2.03	8.349	16.813	**0.001**	37.58 ± 1.71	9.99	21.00	**0.000**	33.83 ± 1.91	7.69	18.52	**0.000**
	FB		49.66 ± 2.24				47.57 ± 2.16				41.52 ± 2.65			
	DIBH	V10Gy [%]	21.21 ± 1.64	6.384	23.134	**0.001**	22.79 ± 1.3	7.49	24.74	**0.000**	22.62 ± 1.22	5.69	20.09	**0.000**
	FB		27.6 ± 1.92				30.28 ± 1.89				28.31 ± 2.0			
	DIBH	V20Gy [%]	5.33 ± 0.39	1.81	25.35	**0.002**	5.82 ± 0.50	2.09	26.42	**0.000**	6.46 ± 0.52	2.22	25.58	**0.021**
	FB		7.14 ± 0.69				7.91 ± 0.78				8.68 ± 0.88			
	DIBH	V25Gy [%]	1.47 ± 0.11	0.636	30.171	**0.003**	1.48 ± 0.10	0.76	33.95	**0.000**	1.32 ± 0.14	0.44	25.13	**0.001**
	FB		2.11 ± 0.23				2.24 ± 0.22				1.76 ± 0.24			
HEART	DIBH	Mean (Gy)	4.96 ± 0.64	2.101	29.738	**0.000**	5.07 ± 0.63	2.54	33.37	**0.000**	5.74 ± 0.73	2.38	29.29	**0.000**
	FB		7.07 ± 0.58				7.61 ± 0.63				8.11 ± 0.69			
	DIBH	V5Gy [%]	27.96 ± 4.08	9.130	24.613	**0.002**	26.39 ± 3.28	12.86	32.76	**0.000**	28.97 ± 4.02	12.91	30.82	**0.000**
	FB		37.09 ± 3.19				39.25 ± 3.58				41.87 ± 4.05			
	DIBH	V10Gy [%]	19.13 ± 3.10	7.382	27.849	**0.003**	18.52 ± 2.58	11.57	38.45	**0.000**	22.54 ± 3.41	10.25	31.25	**0.000**
	FB		26.51 ± 2.52				30.09 ± 2.71				32.78 ± 3.01			
	DIBH	V15Gy [%]	14.14 ± 2.49	6.253	30.658	**0.002**	14.21 ± 2.22	9.49	40.05	**0.000**	17.54 ± 2.67	8.39	32.36	**0.000**
	FB		20.4 ± 2.19				23.7 ± 2.31				25.93 ± 2.55			
BREAST Left	DIBH	Mean (Gy)	5.15 ± 0.42	0.744	12.623	0.209	4.52 ± 0.37	0.86	15.97	0.092	4.44 ± 0.39	0.25	5.43	0.361
	FB		5.89 ± 0.60				5.38 ± 0.56				4.70 ± 0.36			
	DIBH	V4Gy [%]	48.70 ± 5.91	4.201	7.942	0.588	38.23 ± 3.16	5.79	13.15	0.079	27.14 ± 2.54	1.88	6.47	0.269
	FB		52.90 ± 5.09				44.02 ± 4.05				29.02 ± 2.44			
	DIBH	V10Gy [%]	13.16 ± 1.39	6.481	32.993	0.331	14.97 ± 1.63	7.92	34.61	**0.024**	20.42 ± 2.01	1.82	8.17	0.272
	FB		19.65 ± 3.88				22.89 ± 3.54				22.24 ± 2.02			
BREAST Right	DIBH	Mean (Gy)	4.23 ± 0.33	0.714	14.457	0.245	3.63 ± 0.41	0.84	18.68	**0.004**	3.26 ± 0.34	0.32	8.90	0.170
	FB		4.94 ± 0.49				4.47 ± 0.53				3.57 ± 0.38			
	DIBH	V4Gy [%]	42.38 ± 5.38	5.223	10.972	0.523	33.71 ± 4.61	5.02	12.97	0.056	21.59 ± 2.52	1.82	7.75	0.295
	FB		47.60 ± 4.63				38.73 ± 4.23				23.41 ± 2.81			
	DIBH	V10Gy [%]	8.19 ± 0.97	5.437	39.912	**0.030**	12.13 ± 1.84	4.75	28.14	**0.011**	15.78 ± 1.94	1.26	7.37	0.551
	FB		13.62 ± 3.08				16.88 ± 3.02				17.04 ± 2.15			
Integral Dose	DIBH		57,182.72 ± 3631.59	−3088.07	−5.71	0.128	54,898.85 ± 3355.85	−1526.98	−2.86	0.376	52,784.58 ± 3314.10	−2361.46	−4.68	0.160
	FB		54,094.65 ± 3404.70				53,371.87 ± 3131.36				50,423.12 ± 3153.61			

The bold parameters indicate the data is statistically significant (*p*-value < 0.05).

## Data Availability

The data presented in this study are available with the principal Investigator (Dr. J.S.G.), However, due to institutional ethical restrictions of data transfer the anonymized raw data can be shared only on request to the Principal Investigator.

## Supplementary Materials

The following supporting information can be downloaded at: https://www.mdpi.com/article/10.3390/cancers16040690/s1, Table S1 shows the heat map of various dosimetric indices of the target volume and the OARs comparing DIBH with the free breathing method for the three different radiotherapy delivery. Table S2A Shows the dosimetric indices of the OARs comparing three different delivery techniques with DIBH. Table S2B Shows the dosimetric indices of the OARs comparing three different delivery techniques with the Free Breathing method.

## References

[1] Wirth A., Mikhaeel N.G., Aleman B.M., Pinnix C.C., Constine L.S., Ricardi U., Illidge T.M., Eich H.T., Hoppe B.S., Dabaja B. (2020). Involved Site Radiation Therapy in Adult Lymphomas: An Overview of International Lymphoma Radiation Oncology Group Guidelines. Int. J. Radiat. Oncol. Biol. Phys..

[2] Sasse S., Bröckelmann P.J., Goergen H., Plütschow A., Müller H., Kreissl S., Buerkle C., Borchmann S., Fuchs M., Borchmann P. (2017). Long-term follow-up of contemporary treatment in early-stage Hodgkin lymphoma: Updated analyses of the German Hodgkin Study Group HD7, HD8, HD10, and HD11 Trials. J. Clin. Oncol..

[3] Schaapveld M., Aleman B.M., van Eggermond A.M., Janus C.P., Krol A.D., van der Maazen R.W., Roesink J., Raemaekers J.M.M., de Boer J.P., Zijlstra J.M. (2015). Second Cancer Risk Up to 40 Years after Treatment for Hodgkin’s Lymphoma. N. Engl. J. Med..

[4] Hodgson D.C. (2011). Late effects in the era of modern therapy for Hodgkin lymphoma. Hematol. Am. Soc. Hematol. Educ. Program.

[5] Specht L., Yahalom J., Illidge T., Berthelsen A.K., Constine L.S., Eich H.T., Girinsky T., Hoppe R.T., Mauch P., Mikhaeel N.G. (2014). Modern radiation therapy for Hodgkin lymphoma: Field and dose guidelines from the international lymphoma radiation oncology group (ILROG). Int. J. Radiat. Oncol. Biol. Phys..

[6] Maraldo M.V., Aznar M.C., Vogelius I.R., Petersen P.M., Specht L. (2013). Involved node radiation therapy: An effective alternative in early-stage Hodgkin lymphoma. Int. J. Radiat. Oncol. Biol. Phys..

[7] Laskar S., Kumar D.P., Khanna N., Menon H., Sengar M., Arora B., Gujral S., Shet T., Sridhar E., Rangarajan V. (2014). Radiation therapy for early-stage unfavorable Hodgkin lymphoma: Is dose reduction feasible?. Leuk. Lymphoma.

[8] Pern V., Zefkili S., Peurin D., Fourquet A., Kirova Y. (2014). Can we reduce the toxicity of the mediastinal irradiation using new highly conformal techniques?. J. Leuk..

[9] Paumier A., Khodari W., Beaudre A., Ghalibafian M., Blanchard P., Al Hamokles H., Bhari M., Lessard N., Girinsky T. (2011). Intensity-modulated radiotherapy and involved-node concept in patients with Hodgkin lymphoma: Experience of the Gustave-Roussy Institute. Cancer Radiother. J. Soc. Fr. Radiother. Oncol..

[10] Voong K.R., McSpadden K., Pinnix C.C., Shihadeh F., Reed V., Salehpour M.R., Arzu I., Wang H., Hodgson D., Garcia J. (2014). Dosimetric advantages of a “butterfly” technique for intensity-modulated radiation therapy for young female patients with mediastinal Hodgkin’s lymphoma. Radiat. Oncol..

[11] Buglione M., Guerini A.E., Filippi A.R., Spiazzi L., Pasinetti N., Magli A., Toraci C., Borghetti P., Triggiani L., Alghisi A. (2021). A Systematic Review on Intensity Modulated Radiation Therapy for Mediastinal Hodgkin’s Lymphoma. Crit. Rev. Oncol./Hematol..

[12] Fiandra C., Filippi A.R., Catuzzo P., Botticella A., Ciammella P., Franco P., Borca V.C., Ragona R., Tofani S., Ricardi U. (2012). Different IMRT solutions vs. 3D-Conformal Radiotherapy in early-stage Hodgkin’s lymphoma: Dosimetric comparison and clinical considerations. Radiat. Oncol..

[13] Ong C., Verbakel W.F., Cuijpers J.P., Slotman B.J., Senan S. (2011). Dosimetric impact of interplay effect on RapidArc lung stereotactic treatment delivery. Int. J. Radiat. Oncol. Biol. Phys..

[14] Berbeco R.I., Pope C.J., Jiang S.B. (2006). Measurement of the interplay effect in lung IMRT treatment using EDR2 films. J. Appl. Clin. Med. Phys..

[15] Ferini G., Molino L., Tripoli A., Valenti V., Illari S.I., Marchese V.A., Cravagno I.R., Borzi G.R. (2021). Anatomical Predictors of Dosimetric Advantages for Deep-inspiration-breath-hold 3D-conformal Radiotherapy Among Women with Left Breast Cancer. Anticancer Res..

[16] Ferini G., Valenti V., Viola A., Umana G.E., Martorana E. (2022). A Critical Overview of Predictors of Heart Sparing by Deep-Inspiration-Breath-Hold Irradiation in Left-Sided Breast Cancer Patients. Cancers.

[17] Houlihan O.A., Rangaswamy G., Dunne M., Rohan C., O’Neill L., Chalke S., Daly P., Gillham C., McArdle O. (2021). Deep inspiration breath hold versus free breathing technique in mediastinal radiotherapy for lymphoma. BJR Open.

[18] Petersen P.M., Aznar M.C., Berthelsen A.K., Loft A., Schut D.A., Maraldo M., Josipovic M., Klausen T.L., Andersen F.L., Specht L. (2015). Prospective phase II trial of image-guided radiotherapy in Hodgkin lymphoma: Benefit of deep inspiration breath-hold. Acta Oncol..

[19] Illidge T., Specht L., Yahalom J., Aleman B., Berthelsen A.K., Constine L., Dabaja B., Dharmarajan K., Ng A., Ricardi U. (2014). Modern radiation therapy for nodal non-Hodgkin lymphoma—Target definition and dose guidelines from the International Lymphoma Radiation Oncology Group. Int. J. Radiat. Oncol. Biol. Phys..

[20] Kataria T., Sharma K., Subramani V., Karrthick K.P., Bisht S.S. (2012). Homogeneity Index: An objective tool for assessment of conformal radiation treatments. J. Med. Phys..

[21] Wu Q., Mohan R., Morris M., Lauve A., Schmidt-Ullrich R. (2003). Simultaneous integrated boost intensity-modulated radiotherapy for locally advanced head-and-neck squamous cell carcinomas. I: Dosimetric results. Int. J. Radiat. Oncol. Biol. Phys..

[22] Younge K.C., Roberts D., Janes L.A., Anderson C., Moran J.M., Matuszak M.M. (2016). Predicting deliverability of volumetric-modulated arc therapy (VMAT) plans using aperture complexity analysis. J. Appl. Clin. Med. Phys..

[23] Aoyama H., Westerly D.C., Mackie T.R., Olivera G.H., Bentzen S.M., Patel R.R., Jaradat H., Tome W.A., Ritter M.A., Mehta M.P. (2006). Integral radiation dose to normal structures with conformal external beam radiation. Int. J. Radiat. Oncol. Biol. Phys..

[24] Everett A.S., Hoppe B.S., Louis D., McDonald A.M., Morris C.G., Mendenhall N.P., Li Z., Flampouri S. (2019). Comparison of Techniques for Involved-Site Radiation Therapy in Patients with Lower Mediastinal Lymphoma. Pract. Radiat. Oncol..

[25] Starke A., Bowden J., Lynn R., Hall K., Hudson K., Rato A., Aldridge E., Robb D., Steele P., Brady J. (2018). Comparison of butterfly volumetric modulated arc therapy to full arc with or without deep inspiration breath hold for the treatment of mediastinal lymphoma. Radiother. Oncol..

[26] Dores G.M., Metayer C., Curtis R.E. (2002). Second malignant neoplasms among long-term survivors of Hodgkin’s disease: A population-based evaluation over 25 years. J. Clin. Oncol..

[27] Aleman B.M., van den Belt-Dusebout A.W., Klokman W.J., van’t Veer M.B., Bartelink H., van Leeuwen F.E. (2003). Long-term cause-specific mortality of patients treated for Hodgkin’s disease. J. Clin. Oncol..

[28] Filippi A.R., Ragona R., Fusella M., Botticella A., Fiandra C., Ricardi U. (2013). Changes in breast cancer risk associated with different volumes, doses, and techniques in female Hodgkin’s lymphoma patients treated with supra-diaphragmatic radiotherapy. Pract. Radiat. Oncol..

[29] Begosh-Mayne D., Kumar S.S., Toffel S., Okunieff P., O’Dell W. (2020). The dose-response characteristics of four NTCP models: Using a novel CT-based radiomic method to quantify radiation-induced lung density changes. Sci. Rep..

[30] Marks L.B., Yorke E.D., Jackson A., Ten Haken R.K., Constine L.S., Eisbruch A., Bentzen S.M., Nam J., Deasy J.O. (2010). Use of normal tissue complication probability models in the clinic. Int. J. Radiat. Oncol. Biol. Phys..

[31] Palma G., Monti S., Conson M., Pacelli R., Cella L. (2019). Normal tissue complication probability (NTCP) models for modern radiation therapy. Semin. Oncol..

[32] Aznar M.C., Maraldo M.V., Schut D.A., Lundemann M., Brodin N.P., Vogelius I.R., Berthelsen A.K., Specht L., Petersen P.M. (2015). Minimizing late effects for patients with mediastinal Hodgkin lymphoma: Deep inspiration breath-hold, IMRT, or both?. Int. J. Radiat. Oncol. Biol. Phys..

