# Supplementary material for: Dosimetric Impact of Voluntary Deep Inspiration Breath Hold (DIBH) in Mediastinal Hodgkin Lymphomas: A Comparative Evaluation of Three Different Intensity Modulated Radiation Therapy (IMRT) Delivery Methods Using Voluntary DIBH and Free Breathing Techniques
Source: Cancers (Basel). 2024 Feb 6;16(4):690. doi: 10.3390/cancers16040690 (PMC10886974; doi:10.3390/cancers16040690)
Supplement: Supplementary file 1 [file cancers-16-00690-s001.zip › cancers-2703817-supplementary.pdf]

Supplementary Tables

Supplementary Table S1 shows the heat map of various dosimetric indices of the target volume and the OARs comparing DIBH with the free breathing method for the three different radiotherapy delivery

		F-VMAT		B-VMAT		FF-IMRT	
		**DIBH**	**FB**	**DIBH**	**FB**	**DIBH**	**FB**
PTV	Coverage	95.62	95.71	95.44	95.59	95.67	95.68
LUNG Left	Mean (Gy)	5.64	6.73	5.57	7.01	5.33	6.53
	V5Gy[%]	41.17	46.94	37.98	46.97	33.09	40.81
	V10Gy[%]	22.16	28.14	22.77	30.64	22.43	27.54
	V25Gy[%]	1.53	2.20	1.57	2.36	1.49	1.91
LUNG Right	Mean (Gy)	5.74	7.01	5.59	7.11	5.40	6.78
	V5Gy[%]	42.76	52.20	38.08	48.67	34.23	43.01
	V10Gy[%]	21.71	27.21	22.49	30.19	22.86	29.10
	V25Gy[%]	1.36	2.08	1.43	2.11	1.20	1.66
LUNGS	Mean (Gy)	5.69	6.87	5.58	7.05	5.36	6.66
	V5Gy[%]	41.31	49.66	37.58	47.57	33.83	41.52
	V10Gy[%]	21.21	27.60	22.79	30.28	22.62	28.31
	V25Gy[%]	1.47	2.11	1.48	2.24	1.32	1.76
HEART	Mean (Gy)	4.96	7.07	5.07	7.61	5.74	8.11
	V5Gy[%]	27.96	37.09	26.39	39.25	28.97	41.88
	V10Gy[%]	19.13	26.51	18.52	30.09	22.54	32.78
	V15Gy[%]	14.14	20.40	14.21	23.70	17.54	25.93
BREAST Left	Mean (Gy)	5.15	5.89	4.52	5.38	4.44	4.70
	V4Gy[%]	48.70	52.90	38.23	44.02	27.14	29.02
	V10Gy[%]	13.16	19.65	14.97	22.89	20.42	22.24
BREAST Right	Mean (Gy)	4.23	4.94	3.63	4.47	3.26	3.57
	V4Gy[%]	42.38	47.60	33.71	38.73	21.59	23.41
	V10Gy[%]	8.19	13.62	12.13	16.88	15.78	17.04

F-VMAT: Full Arc Volumetric Arc Therapy; B-VMAT: Butterfly Volumetric Arc Therapy; FF-IMRT: Fixed field Intensity Modulated Radiotherapy

Supplementary Table-S2A. Shows the dosimetric indices of the OARs comparing three different delivery techniques with DIBH

Oargan's At Risk	F-VMAT DIBH		B-VMAT DIBH		FF-IMRT DIBH		Between Groups P-value
Dosimetry	B-VMAT DIBH	FF-IMRT DIBH	F-VMAT DIBH	FF-IMRT DIBH	F-VMAT DIBH	B-VMAT DIBH	
Lung_L Mean Gy	1.000	0.887	1.000	0.699	0.887	0.699	0.428
Lung_L_V5Gy	0.694	0.010	0.694	0.205	0.010	0.205	0.012
Lung_L_V10Gy	1.000	1.000	1.000	1.000	1.000	1.000	0.966
Lung_L_V25Gy	1.000	1.000	1.000	1.000	1.000	1.000	0.938
Lung_R_Mean Gy	1.000	1.000	1.000	1.000	1.000	1.000	0.732
Lung_R_V5Gy	0.433	0.027	0.433	0.683	0.027	0.683	0.032
Lung_R_V10Gy	1.000	1.000	1.000	1.000	1.000	1.000	0.867
Lung_R_V25Gy							0.705
Lungs Mean Gy	1.000	1.000	1.000	1.000	1.000	1.000	0.640
Lungs_V5Gy	0.504	0.020	0.504	0.493	0.020	0.493	0.025
Lungs_V10Gy	1.000	1.000	1.000	1.000	1.000	1.000	0.684
Lungs_V25 Gy	1.000	1.000	1.000	1.000	1.000	1.000	0.565
Heart_Mean Gy*							0.545
Heart_V5Gy*							0.898
Heart_V10Gy*							0.546
Heart_V15Gy*							0.400
Breast_L Mean Gy	1.000	0.991	1.000	1.000	0.991	1.000	0.561
Breast_L_V4Gy*							0.074
Breast_L_V10Gy*							0.108
Breast_R Mean Gy*							0.140
Breast_R_V4Gy	1.000	0.033	1.000	0.262	0.033	0.262	0.035
Breast_R_V10 Gy	0.671	0.026	0.671	0.483	0.026	0.483	0.032
Thyroid Mean Gy*							0.954
Monitor Units (MU)	1.000	<0.05	1.000	<0.05	<0.05	<0.05	<0.05
Homogeneity Index (HI)	0.045	1.000	0.045	0.083	1.000	0.083	0.028
Conformity Index (CI)	0.374	<0.05	0.374	<0.05	<0.05	<0.05	<0.05

Shapiro-Wilk's test was used to check the normality of the dataset. If the P value of Shapiro-Wilk's test was P> 0.05, the data was considered to be normally distributed and a parametric test i.e. one-way ANOVA test was used to compare all three different treatment techniques. If the P value of Shapiro-Wilk's test was P<0.05, the distribution of the data was not considered to be normal and a non-parametric Kruskal-Wallis test was used.
*In the normality check, it was observed that Lung_L Mean Gy, Lung_L_V5Gy, Lung_L_V10Gy, Lung_L_V25Gy, Lung_R_Mean Gy, Lung_R_V5Gy, Lung_R_V10Gy, Lungs_Mean Gy, Lungs_V5Gy, Lungs_V10Gy, Lungs_V25Gy Breast_L_MeanGy, Homogenity Index were normally distributed variables, so for those variables ANOVA test was used and pairwise comparison was done by Bonferroni's Correction.

Significant P values were highlighted. Variables (Lung_R_V25Gy, Heart_Mean Gy, Heart_V5Gy, Heart_V10Gy, Heart_V15Gy, Breast_L_V4Gy, Breast_L_V10Gy, Breast_R_Mean Gy, Breast_R_V4Gy, Breast_R_V10Gy, Thyroid Mean Gy, Monitor Units, Conformity Index) were not normally distributed variables, So for these variables, Kruskal Walli's test was used and pairwise comparison was done only for significant variables (Breast_R_V4Gy, Breast_R_V10Gy, Monitor Units, Conformity Index). Significant P values are highlighted. Kruskal Walli's test does not provide pairwise comparisons if the overall p-value is not significant, hence the table does not show the p-values.

Supplementary Table-S2B. Shows the dosimetric indices of the OARs comparing three different delivery techniques with the Free Breathing method

Oargan's At Risk Dosimetry	F-VMAT FB		B-VMAT FB		FF-IMRT FB		Between Groups P value
	B-VMAT FB	FF-IMRT FB	F-VMAT FB	FF-IMRT FB	F-VMAT FB	B-VMAT FB	
Lungs_Mean Gy	1.000	0.093	1.000	0.020	0.093	0.020	0.017
Lung_L_V5Gy	1.000	0.125	1.000	0.123	0.125	0.123	0.063
Lung_L_V10Gy	1.000	1.000	1.000	0.957	1.000	0.957	0.325
Lung_L_V25Gy	1.000	1.000	1.000	0.725	1.000	0.725	0.492
Lung_R_Mean Gy*							0.738
Lung_R_V5Gy*							0.061
Lung_R_V10Gy*							0.620
Lung_R_V25Gy*							0.333
Lungs_Mean Gy*							0.594
Lungs_V5Gy	1.000	0.053	1.000	0.224	0.053	0.224	0.047
Lungs_V10Gy*							0.492
Lungs_V25Gy	1.000	0.880	1.000	0.444	0.880	0.444	0.325
Heart_Mean Gy	1.000	0.739	1.000	1.000	0.739	1.000	0.508
Heart_V5Gy	1.000	1.000	1.000	1.000	1.000	1.000	0.648

Heart_V10Gy	1.000	0.335	1.000	1.000	0.335	1.000	0.277
Heart_V15Gy	0.977	0.305	0.977	1.000	0.305	1.000	0.255
Breast_L_Mean* Gy							0.622
Breast_L_V4Gy	1.000	0.018	1.000	0.095	0.018	0.095	0.015
Breast_L_V10Gy*							0.308
Breast_R_Mean* Gy							0.115
Breast_R_V4Gy	0.950	0.003	0.950	0.061	0.003	0.061	0.003
Breast_R_V10Gy*							0.115
Thyroid Mean Gy*							0.995
Monitor Units (MU)	0.053	<0.05	0.053	<0.05	<0.05	<0.05	0.000
Homogeneity Index (HI)	0.300	1.000	0.300	0.171	1.000	0.171	0.119
Conformity Index (CI)	0.002	<0.05	0.002	0.046	<0.05	0.046	0.000

Shapiro-Wilk's test was used to check the normality of the dataset. If the P value of Shapiro-Wilk's test was P> 0.05, the data was considered to be normally distributed and a parametric test i.e. one-way ANOVA test was used to compare all three different treatment techniques. If the P value of Shapiro-Wilk's test was P<0.05, the distribution of the data was not considered to be normal and a non-parametric Kruskal-Wallis test was used.

*In the normality check, it was observed that Lung_L Mean Gy, Lung_L_V5Gy, Lung_L_V10Gy, Lung_L_V25Gy, Lungs_V5Gy, Lungs_V25Gy, HI, Heart_Mean Gy, Heart_V5Gy, Heart_V10Gy, Heart_V15Gy were normally distributed variables, so for those variables ANOVA test was used and pairwise comparison was done by Bonferroni's Correction. Significant p- p-values are highlighted. Variables (Lung_R_Mean Gy, Lung_R_V5Gy, Lung_R_V10Gy, Lung_R_V25Gy, Lungs_Mean Gy, Lungs_V10Gy, Breast_L_MeanGy, Breast_L_V4Gy, Breast_L_V10Gy, Breast_R_Mean Gy, Breast_R_V4Gy, Breast_R_V10Gy, Thyroid Mean Gy, Monitor Units) were not normally distributed, so for these variables Kruskal Walli's test was used and pairwise comparison was done only for significant variables. (Breast_L_V4Gy, Breast_R_V10Gy, Monitor Units,). Significant P values were highlighted. Kruskal Walli's test does not provide pairwise comparisons if the overall p-value is not significant, hence the table does not show the p-values.
